# Atomistic Kinetics of Dislocation‐Mediated Grain Growth in Monolayer MoS_2_

**DOI:** 10.1002/advs.77465

**Published:** 2026-08-30

**Authors:** Chang‐Won Choi, Min‐Yeong Choi, Tao Du, Jinho Rhee, Seokho Moon, Yeon‐Seo Nam, Xiaoyan Lu, Jong Kyu Kim, Jungwon Park, Moon‐Ho Jo, Cheol‐Joo Kim, Si‐Young Choi

**Affiliations:** ^1^ Center for Van der Waals Quantum Solids Institute for Basic Science (IBS) Pohang Republic of Korea; ^2^ Department of Materials Science and Engineering Pohang University of Science and Technology (POSTECH) Pohang Republic of Korea; ^3^ Department of Chemical Engineering POSTECH Pohang Republic of Korea; ^4^ School of Civil Engineering Harbin Institute of Technology Harbin China; ^5^ Department of Chemical and Biological Engineering Seoul National University (SNU) Seoul Republic of Korea; ^6^ Department of Materials Science and Engineering Ajou University Suwon Republic of Korea; ^7^ Department of Energy Systems Research Ajou University Suwon Republic of Korea; ^8^ Department of Physics POSTECH Pohang Republic of Korea; ^9^ Department of Semiconductor Engineering POSTECH Pohang Republic of Korea

**Keywords:** 2D materials, dislocation, grain boundary, in‐situ STEM

## Abstract

Grain boundaries (GBs) in 2D materials are arrays of dislocations that strongly influence electronic, optical, mechanical, and transport properties. In monolayer MoS_2_, Mo 5|7 and S 5|7 dislocation cores are structurally established, yet how their distinct mobilities govern GB migration and grain growth has remained unresolved. Here, we directly image and quantify GB mobility in polycrystalline monolayer MoS_2_ at the level of individual dislocation cores. An atomic‐scale in‐situ heating study reveals that mobile GBs consist solely of Mo 5|7 dislocations, whereas immobile GBs contain S 5|7 kink sites that suppress GB motion. Dark‐field TEM grain‐growth measurements show that mobile GBs drive rapid coarsening, while S 5|7‐pinned immobile GBs arrest microstructural evolution. Molecular dynamics simulations correlate this mobility contrast with distinct local mechanical and energetic environments around Mo 5|7 and S 5|7 cores, providing an atomistic origin for their different migration kinetics. These findings establish a direct structure‐kinetics‐growth relationship for GB evolution in monolayer MoS_2_ and demonstrate a broadly applicable in‐situ electron‐microscopy platform for probing defect‐driven microstructural dynamics in 2D materials.

## Graphical abstract

Atomic‐resolution in‐situ heating microscopy directly visualizes dislocation‐mediated grain boundary migration and grain growth in monolayer MoS_2_. Mobile grain boundaries migrate through collective motion of Mo 5|7 dislocations, whereas S 5|7 defects remain largely immobile. Complementary atomistic simulations reveal the atomic origin of this contrasting mobility, providing mechanistic insight into grain growth in 2D materials.

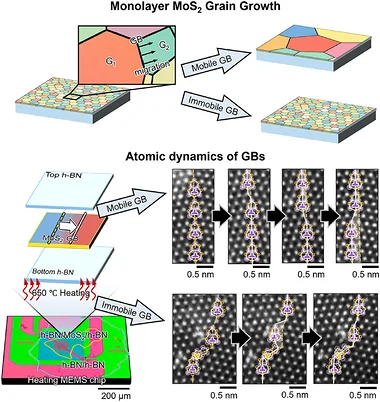

## 1 Introduction

Polycrystalline monolayer MoS_2_ consists of misoriented grains separated by grain boundaries (GBs) [1, 2, 3, 4, 5, 6, 7, 8, 9], which locally break lattice continuity and strongly modify the structural, electronic, optical, and mechanical properties of the crystal [10, 11, 12, 13]. In this system, GBs are primarily constructed from elemental 5|7 dislocation cores with the smallest Burgers vector, most notably the Mo 5|7 and S 5|7 configurations defined by Mo─Mo and S─S homoelemental bonds at the shared 5|7 ring edge, respectively [1, 5, 14, 15]. Because such defects are unavoidable in large‐area chemical‐vapor‐deposited MoS_2_, understanding how their atomic structures influence mesoscale microstructural evolution is essential for defect engineering and for practical control of material performance [16, 17, 18, 19, 20, 21, 22, 23, 24].

Previous studies have focused largely on the thermodynamic formation, structural identification, and electronic consequences of GB defects in MoS_2_ [10, 11, 12, 13, 25, 26, 27, 28]. These studies established that Mo─Mo and S─S homoelemental bonds in 5|7 dislocation cores perturb the local coordination and bond strain [29], thereby giving rise to distinct defect‐induced electronic states. In parallel, density functional theory calculations predicted markedly different migration barriers for the two representative dislocation cores, with a relatively low gliding barrier for Mo 5|7 and a much higher barrier for S 5|7 [30]. Other first‐principles studies have shown that mechanical loading can induce dislocation glide and annihilation [31]. These predictions imply that dislocation chemistry should strongly influence GB mobility, but they do not by themselves explain how local bonding, stress concentration, and interactions among neighboring defect units determine real‐time migration behavior under thermal driving. A direct experimental framework that resolves individual dislocation motion while linking it to the surrounding mechanical and energetic environment is therefore needed.

More broadly, GB migration is a dislocation‐mediated kinetic process in which collective atomic rearrangements govern grain growth, coalescence, and defect healing [27, 28, 30]. Although in‐situ electron microscopy has revealed GB motion in several material systems [32, 33, 34], direct atomic‐scale measurements that connect the trajectories of specific dislocation cores to grain growth behavior in beam‐sensitive 2D semiconductors remain limited. This gap is particularly important in monolayer MoS_2_, where the small set of accessible GB core structures provides an ideal platform for establishing a quantitative relationship between defect structure, GB mobility, and microstructural evolution.

Here, we establish such a structure‐kinetics‐growth framework for polycrystalline monolayer MoS_2_. High‐angle annular dark‐field scanning transmission electron microscopy (HAADF‐STEM) identifies the dislocation architectures that distinguish mobile and immobile GBs, while in‐situ heating STEM combined with self‐supervised machine‐learning denoising [35] and automated defect tracking resolves the real‐time migration dynamics of individual Mo 5|7 and S 5|7 defects. Complementary molecular dynamics (MD) simulations reveal the local mechanical and energetic origins of their contrasting mobilities, and dark‐field (DF) TEM links these kinetic differences to the resulting grain growth behavior. Together, these results suggest an atomic‐scale framework for understanding chemistry‐dependent GB migration in binary 2D materials (e.g., h‐BN, WSe_2_, MoSe_2_, and PtSe_2_) [30, 36, 37, 38] and provide a generally useful platform for dynamic defect analysis in low‐dimensional materials.

## 2 Results

**FIGURE 1:**
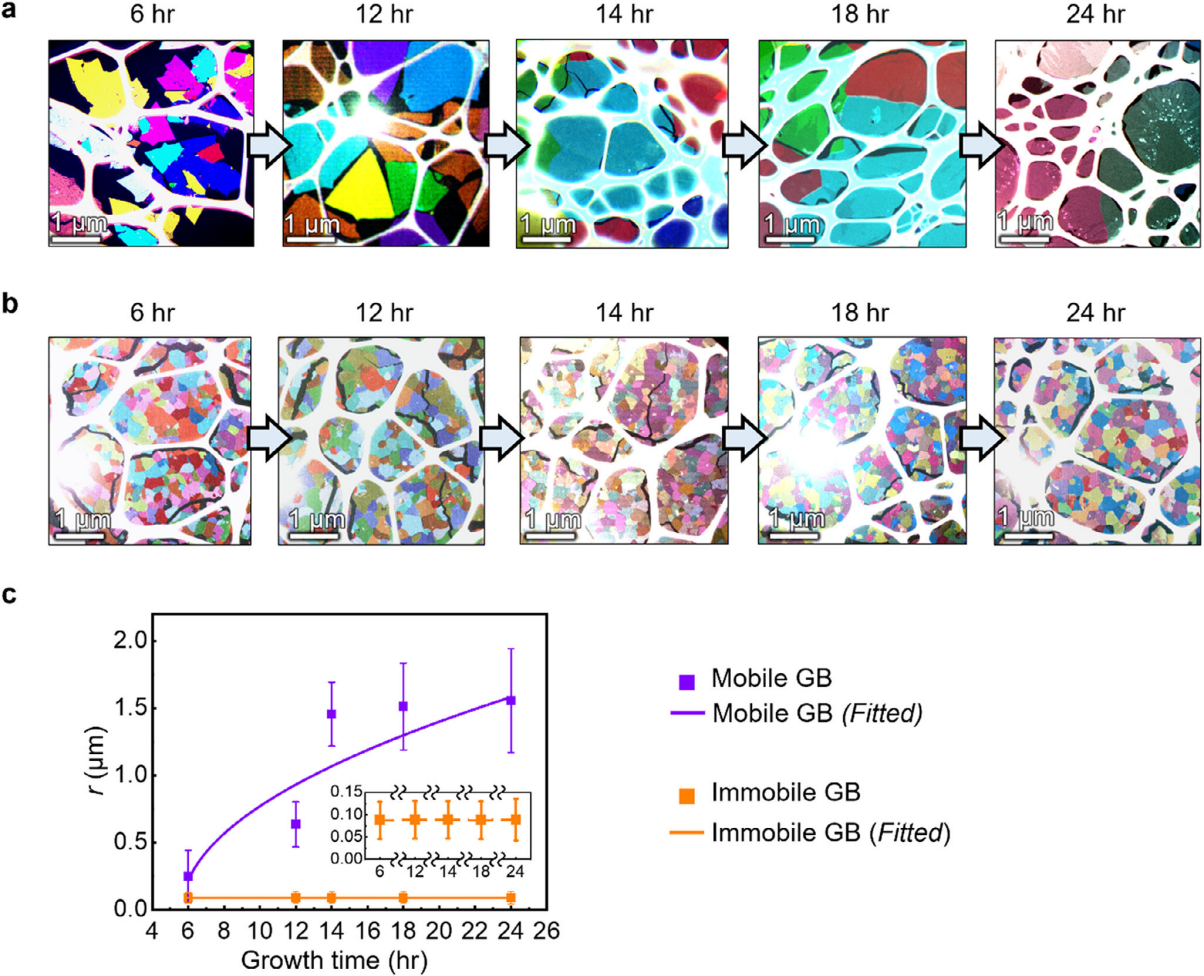
Grain growth behavior of MoS_2_ with mobile and immobile GBs observed by DF‐TEM. (a) DF‐TEM images of MoS_2_ with mobile GBs after growth times of 6, 12, 14, 18, and 24 h. Each color indicates a distinct crystallographic orientation. (b) DF‐TEM images of MoS_2_ with immobile GBs after growth times of 6, 12, 14, 18, and 24 h. (c) Plot of the average grain radius as a function of growth time for MoS_2_ with mobile and immobile GBs. The inset shows an enlarged view of the immobile GB data. Error bars represent standard deviations. The following denote: purple square symbols – mobile GBs; orange square symbols – immobile GBs; purple solid line – fitted curve for mobile GBs; orange solid line – fitted curve for immobile GBs.

To establish the kinetic basis for grain growth in polycrystalline monolayer MoS_2_, we first compared MoS_2_ grown under Mo‐rich and Mo‐poor growth conditions at 650°C [25], which exhibit sharply different microstructural evolution (Figure 1). DF‐TEM was used to track grain evolution from 6 to 24 h, with false‐color contrast assigned according to crystallographic orientation identified by selected‐area electron diffraction (Figure 1a,b and Figure S1). Figure 1a shows that Mo‐rich MoS_2_ undergoes pronounced grain growth, including progressive grain enlargement, grain coalescence, and a substantial reduction in GB density. Because this behavior requires active GB migration and elimination, we define these GBs as mobile GBs. In contrast, Figure 1b shows that the Mo‐poor MoS_2_ remains fine‐grained throughout growth, with little apparent coalescence. Because this stagnant morphology indicates suppressed GB migration, we define these GBs as immobile GBs. Figure 1c quantifies in more detail these distinct growth behaviors through the average grain radius extracted from the DF‐TEM images (Table S1). In mobile GB MoS_2_ grown under Mo‐rich conditions, the average grain radius increases continuously with growth time. This continuous coarsening is consistent with a strong thermodynamic driving force that emerges when neighboring grains impinge, because grain contact maximizes the local interfacial energy and promotes rapid GB migration to reduce the total GB area. Grain growth then continues more gradually, reaching 1.56 µm at 24 h, while the increasing standard deviation indicates continued coarsening and broadening of the grain‐size distribution. In contrast, the immobile GB MoS_2_ under Mo‐poor conditions shows essentially no grain growth throughout the same period: the average grain radius remains approximately 0.088 µm with a standard deviation of 0.042 µm, indicating that GB migration is strongly suppressed. Figure S2 summarizes the grain‐size distributions at different growth times, revealing the progressive grain coarsening of mobile GB MoS_2_ and the nearly unchanged grain‐size distribution of immobile GB MoS_2_. Temperature‐dependent growth experiments further confirmed that 650°C is a suitable temperature for systematically comparing the grain growth behaviors of mobile and immobile MoS_2_ (Figure S3). Together, these results establish that GB mobility provides the kinetic basis for grain growth behavior predicted by theoretical and computational studies [39, 40, 41], with mobile GBs driving rapid grain coarsening and immobile GBs arresting microstructural evolution in monolayer MoS_2_.

**FIGURE 2:**
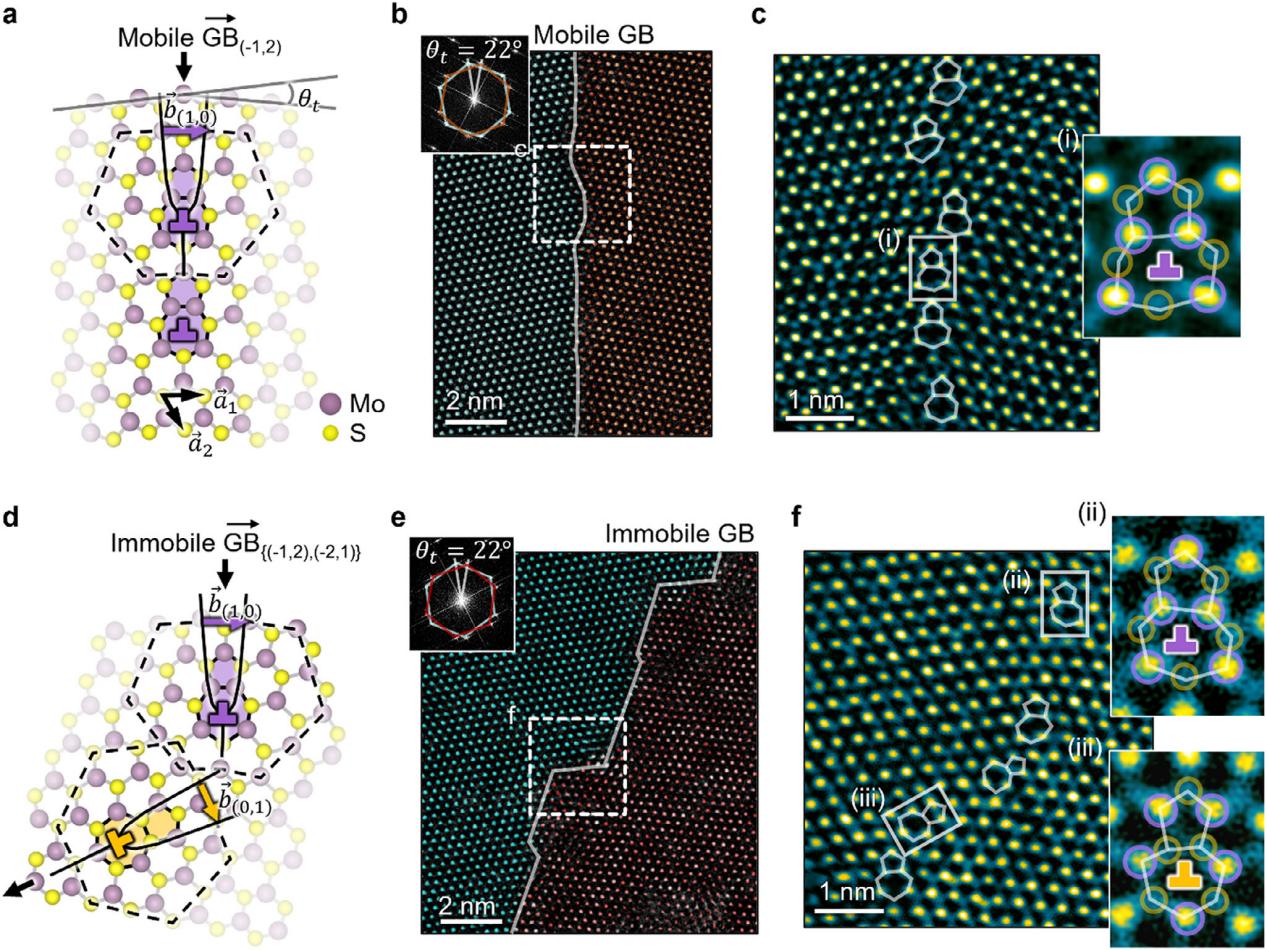
Structural definition of mobile and immobile MoS_2_ GBs. (a) Definition of a mobile GB composed solely of Mo 5|7s, shown in the schematic illustration. The vectors ${\vec{a}}_{1}$ and ${\vec{a}}_{2}$ correspond to (1,0) and (0,1), respectively, and the tilt angle is represented by ***θ***_***t***_. The dashed hexagon indicates the Burgers circuit, and the purple arrow represents the Burgers vector of Mo 5|7 (${\vec{b}}_{\left(1,0\right)}$). The mobile GB vector is ${\vec{\mathrm{GB}}}_{\left(-1,2\right)}$. (b) HAADF–STEM image of a mobile GB composed solely of Mo 5|7 (inset (i)). The G_L_ and G_R_ are false‐colored blue and red, respectively, to distinguish their orientations. A white line indicates the GB. The inset FFT patterns in (b) confirm that ***θ***_***t***_= 22°. (c) High‐magnification HAADF–STEM image of the dashed boxed region in (b). The mobile GB consists of a periodic array of Mo 5|7s, as shown in inset (i). (d) Definition of an immobile GB composed of Mo 5|7 and S 5|7, shown in the schematic illustration. The orange arrow represents the Burgers vector of S 5|7 (${\vec{b}}_{\left(0,1\right)}$). The GB is expressed as a shift from (−1,2) to (−2,1), indicated by the {(−1,2),(−2,1)} notation. (e) HAADF–STEM image of an immobile GB containing mixed Mo 5|7 and S 5|7. The GB is indicated by a white line. (f) High‐magnification HAADF–STEM image of the boxed region in (e). The immobile GB is composed of a non‐periodic array of Mo 5|7 and S 5|7, as shown in insets (ii) and (iii), respectively.

Figure 2 identifies the atomic structures underlying the mobile and immobile GBs defined by their grain growth behavior in Figure 1. The mobile GB consists of a periodic array of Mo 5|7 dislocation cores, as schematically illustrated in Figure 2a. The corresponding false‐colored HAADF‐STEM image in Figure 2b shows a smooth and continuous GB trace with a tilt angle (*θ*_*t*_) of approximately 22°. High‐magnification imaging of the dashed rectangle region further resolves the individual dislocation cores and confirms that the smooth GB is composed exclusively of Mo 5|7 units (Figure 2c). This periodic Mo 5|7 arrangement provides a structurally uniform GB without pronounced kink or pinning sites. In contrast, Figure 2d defines the immobile GB as a mixed dislocation array containing both Mo 5|7 and S 5|7 cores. HAADF‐STEM imaging shows that this GB exhibits a jagged morphology with sharp kinks rather than a smooth GB trace (Figure 2e). Atomic‐resolution analysis reveals that the straight segments are composed of Mo 5|7 cores, whereas S 5|7 cores are located at the kink sites (Figure 2f). The incorporation of S 5|7, therefore, locally changes the GB direction and produces a faceted GB morphology. Because these S 5|7 kink sites interrupt the otherwise continuous Mo 5|7 array, they provide structural pinning centers that can suppress collective GB migration. Together, these observations show that GB mobility in monolayer MoS_2_ is determined not simply by the *θ*_*t*_, but by the dislocation‐core chemistry and its spatial arrangement along the GB. Mobile GBs composed only of Mo 5|7 cores are structurally uniform, whereas immobile GBs containing S 5|7 cores become kinked. Detailed crystallographic definitions of the Burgers vectors, GB vectors, GB planes, and defect compositions are provided in Notes S1 and S2 and Figures S4 and S5. The identified dislocation cores are consistent with the previously established Mo 5|7 and S 5|7 configurations [14, 25, 29, 41, 42].

**FIGURE 3:**
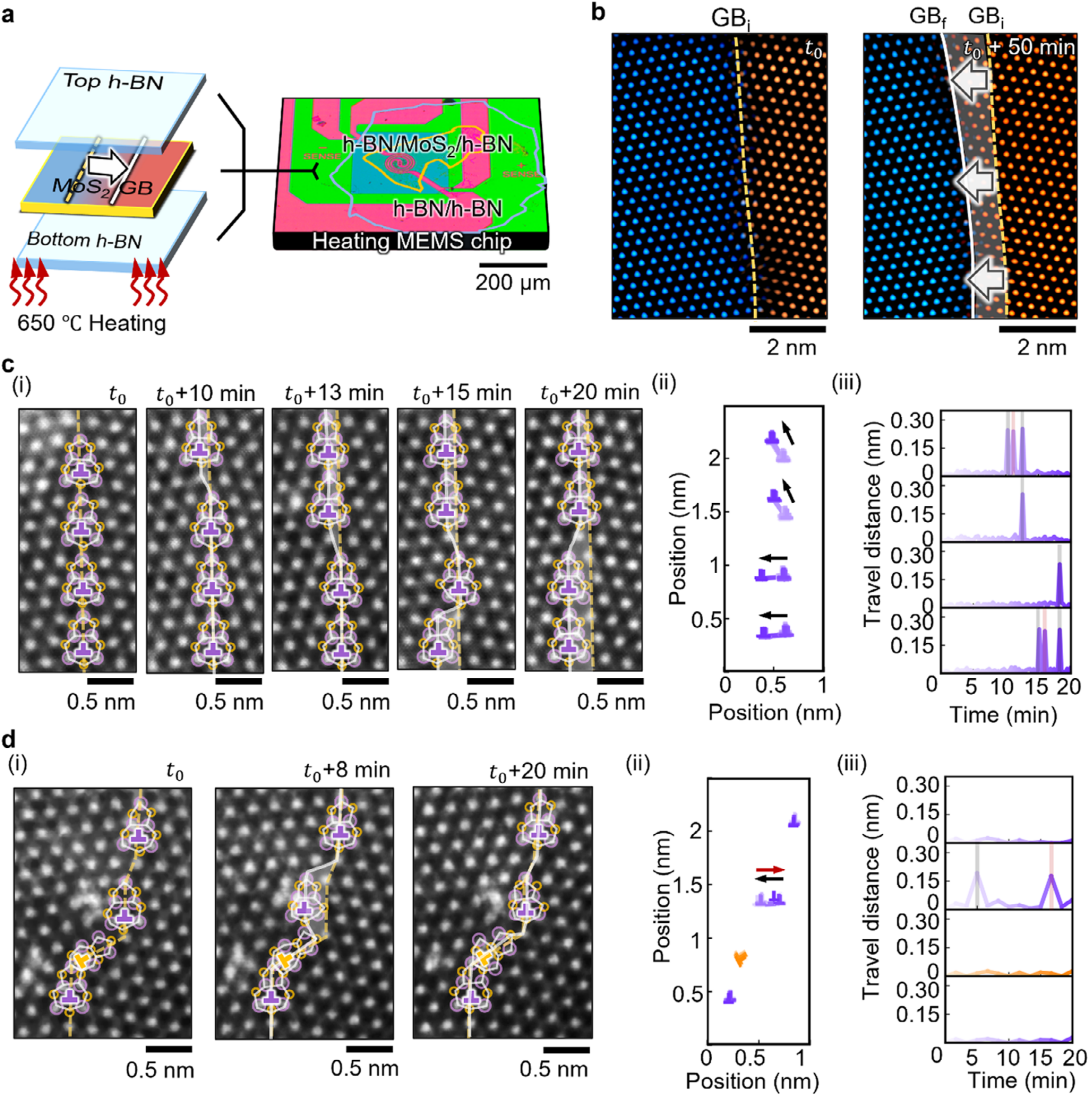
Real‐time in‐situ observation of GB migration in monolayer MoS_2_ during thermal stimulation. (a) Schematic illustration of the in‐situ heating STEM setup. The polycrystalline monolayer MoS_2_ containing GBs was fully encapsulated between top and bottom h‐BN layers and mounted on a MEMS heating chip. (b) False‐colored HAADF‐STEM image obtained from inverse‐FFT analysis, showing the migration of a mobile GB. The dashed yellow line indicates the GB_i_ at ***t***_0_, while the solid white line represents the GB_f_ after 50 min of heating. G_R_ (red) and G_L_ (blue) indicate the grains on either side of the GB. (c) Time‐lapsed HAADF–STEM snapshots of a mobile GB composed solely of Mo 5|7s. (i) The sequential frames (***t***_0_ → ***t***_0_ + 10 min → ***t***_0_ + 13 min → ***t***_0_ + 15 min → ***t***_0_ + 20 min) show progressive migration of individual Mo 5|7s. The purple circles represent Mo atoms, and the orange circles represent S atoms. (ii) The tracking graph of Mo 5|7s, where the black arrows indicate their migration directions. (iii) Absolute distance of individual Mo 5|7s. (d) In‐situ heating STEM movie snapshots of an immobile GB at different time intervals (***t***_0_ → ***t***_0_ + 20 min) shown in (i). (ii) shows the graph tracking the movement of both Mo 5|7 and S 5|7, with arrows indicating the migration directions. (iii) Absolute distance of individual Mo 5|7 and S 5|7. Grey regions indicate forward climbing/gliding, while red regions indicate backward climbing/gliding.

Figure 3 probes the real‐time atomic‐scale kinetics of mobile and immobile GBs by in‐situ heating STEM. Figure 3a represents the experimental platform. Because the observations were conducted at 650°C under an 80 kV accelerating voltage, both types of MoS_2_ microstructures were fully encapsulated between 2 nm‐thick h‐BN layers to enable stable atomic‐resolution imaging while minimizing high‐temperature and beam‐induced instability [43, 44]. Under HAADF conditions (collection semi‐angle of 54–216 mrad), the low‐Z h‐BN layers contribute negligibly to the image contrast, allowing the heavier MoS_2_ lattice to be imaged with high Z‐sensitive contrast. The encapsulating h‐BN is visible only in LAADF imaging (collection semi‐angle of 23–92 mrad), consistent with its weak scattering signal [45]. This encapsulated platform, therefore, enables direct and comparative observation of thermally activated GB dynamics in monolayer MoS_2_ with sufficient structural and imaging stability [46, 47, 48].

Figure 3b,c show the migration dynamics of a mobile GB during in‐situ heating at 650°C, and the time‐lapsed HAADF‐STEM images were processed using the self‐supervised denoising framework [35] to improve atomic‐scale interpretability [49]. The positional deviations relative to the simulated reference were 9.0 ± 5.6 × 10^−3^ nm for the raw image and 6.8 ± 5.4 × 10^−3^ nm for the denoised image, indicating improved atomic‐position accuracy after denoising, with no appreciable systematic directional shift (Figure S6). In Figure 3b, the initial position of the mobile GB (GB_i_) is marked by the yellow dashed line between the blue left grain and the red right grain. After 50 min of heating, the mobile GB migrates, as indicated by the final position (GB_f_) shown by the white solid line. The clear displacement of the interface demonstrates thermally‐activated migration of the GBs. Figure 3ci and Movie S1 resolve the sequential migration of four aligned Mo 5|7 dislocations in the same direction, resulting in collective translation of the GB. After 10 min of annealing (*t*_0_ + 10 min), the uppermost Mo 5|7 migrated first through climbing. At *t*_0_ + 13 min, the second Mo 5|7 moved through climbing as well. The bottommost Mo 5|7 then begins to glide at *t*_0_ + 15 min, and the adjacent Mo 5|7 glides at *t*_0_ + 20 min, resulting in sequential translation of the entire GB. The tracking map in Figure 3cii and the displacement plot in Figure 3ciii show that each Mo 5|7 undergoes intermittent periods of forward and backward motion, but the overall behavior is directional and correlated. Sharp peaks correspond to active migration events, whereas flat regions indicate temporary arrest. The cumulative displacement of the mobile GB shown in Figure S7a confirms that the Mo 5|7 units continue to migrate over time and collectively drive GB translation and further that sequential glide and climb of Mo 5|7 dislocations collectively drive mobile GB migration.

A markedly different kinetic behavior is observed for the immobile GB in Figure 3d and Movie S2. During 20 min of annealing, the S 5|7 core remains completely stationary, whereas only one neighboring Mo 5|7 undergoes a transient glide event at 8 min before returning to its original position by 20 min. No sustained or correlated migration is observed among the other defect units. The tracking analysis in Figure 3dii and the displacement plot in Figure 3diii confirm that the S 5|7 sites do not show detectable movement and that the adjacent Mo 5|7 dislocations exhibit only localized, reversible motion. Accordingly, the cumulative distances of both Mo 5|7 and S 5|7 within the immobile GB are far smaller than those of the mobile GB (Figure S7). These results show that S 5|7 acts as a strong pinning center that interrupts the cooperative propagation of neighboring Mo 5|7 dislocations and immobilizes the interface as a whole. GB mobility in monolayer MoS_2_ is therefore controlled by the local dislocation composition: GBs composed entirely of Mo 5|7 are mobile, whereas incorporation of S 5|7 produces kinetic pinning under identical thermal conditions. Consistent immobile behavior was also observed for S 5|7‐containing GBs at 550°C and 700°C, providing complementary support for the persistence of this pinning behavior under different thermal conditions without determining the activation energy (Figure S8). This behavior illustrates how an immobile defect can suppress collective GB migration by pinning neighboring dislocations [50].

**FIGURE 4:**
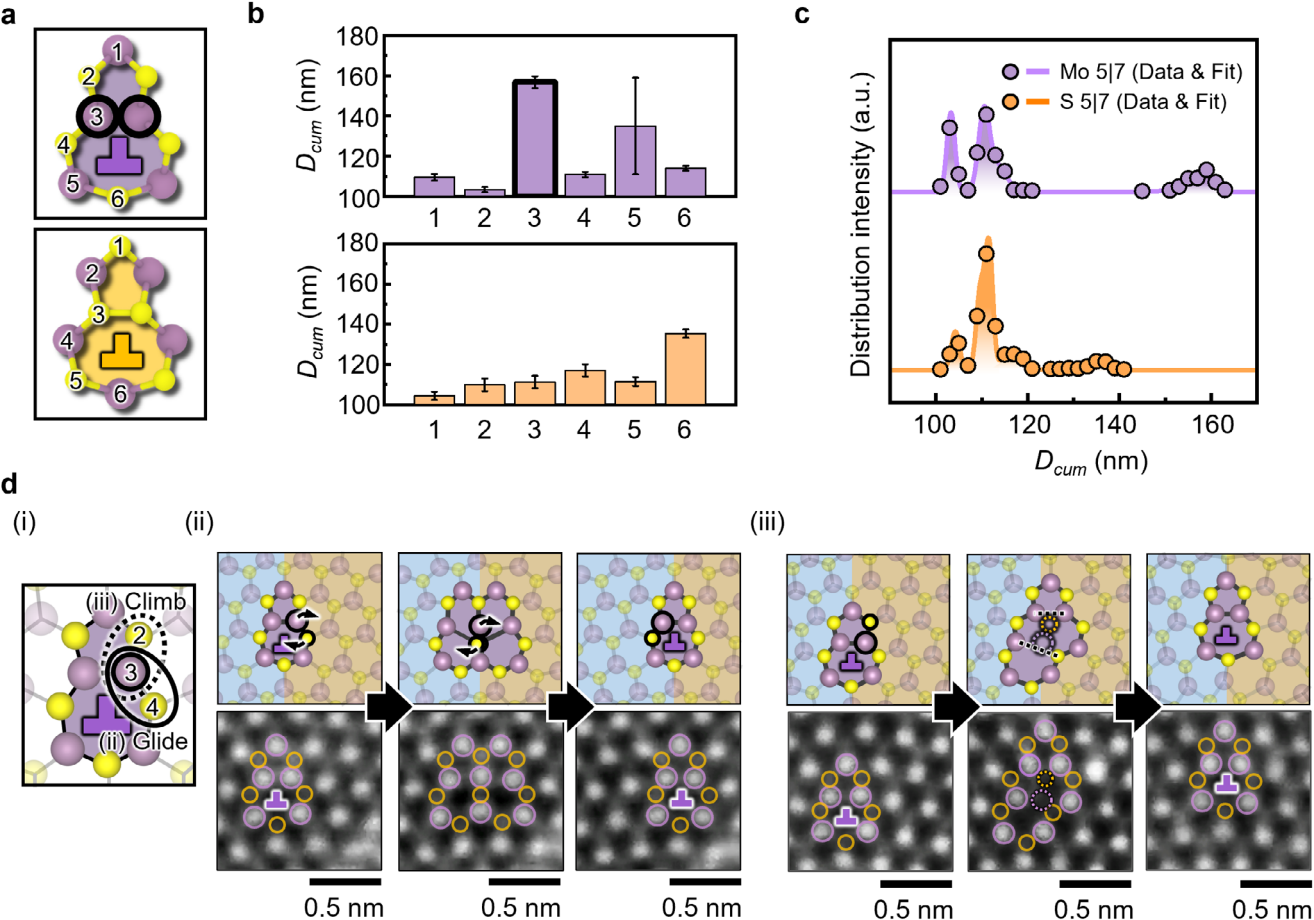
Site‐dependent local dynamic responses of Mo 5|7 and S 5|7 at 923 K by MD simulations. (a) Schematics defining the atomic sites (1–6) within the Mo 5|7 (upper panel) and S 5|7 (lower panel). The highlighted site indicates the atomic positions exhibiting enhanced local dynamic activity. (b) Site‐resolved averaged cumulative non‐affine displacement values (*D*_cum_) for atoms located at different positions within the Mo 5|7 and S 5|7. The upper and lower panels correspond to the Mo 5|7 and S 5|7 dislocation cores, respectively. (c) Distributions of *D*_cum_ for atoms within different GBs after 1 ns of simulation. (d) Atomic pathways for Mo 5|7 migration during (ii) glide and (iii) climb. (i) Schematic illustration of Mo 5|7 migration pathways. Solid and dashed circles denote the sequential coupled motions between sites 3 and 4 (glide) and sites 3 and 2 (climb), respectively. (ii, iii) Atomic models (upper panels) and corresponding horizontally flipped in‐situ STEM snapshots (lower panels) showing glide and climb through coupled processes involving sites 3 and 4 or sites 3 and 2, respectively. Arrows indicate atomic motion, solid circles denote atoms involved in the migration process, dashed circles indicate disappearing atoms, and dashed lines represent formed bonds.

To unveil the atomistic origin of this mobility contrast, MD simulations were used to analyze the site‐dependent local dynamic responses of Mo 5|7 and S 5|7 dislocations (Figure 4). The simulation models are provided in Figure S9, and the computational details are described in the Methods. To investigate the origin of the activated sites governing dislocation migration at elevated temperatures, the simulation temperature was set to 650°C. In the Mo 5|7 core (upper panel of Figure 4a), site 3 corresponds to the Mo atom at the Mo─Mo bonding region and emerges as the most dynamically activated position. By contrast, the S 5|7 core does not contain an equivalently activated internal site, indicating a weaker and more spatially uniform local response (lower panel of Figure 4a). This contrast is evident in the site‐resolved cumulative non‐affine displacement, *D*_cum_, shown in Figure 4b. For Mo 5|7, site 3 exhibits the largest *D*_cum_ value of 156.8 nm, substantially higher than those of site 1 (109.5 nm), site 2 (103.4 nm), site 4 (110.9 nm), site 5 (135.0 nm), and site 6 (114.1 nm). In contrast, site 3 of the S 5|7 core exhibits a *D*_cum_ value of only 111.3 nm, which is not distinctly higher than those of the other S 5|7 sites (104.5–135.4 nm). Figure 4c shows that the broader and higher‐*D*_cum_ distribution of the Mo 5|7 GB contributes to its higher mobility, whereas the lower‐*D*_cum_ distribution of the S 5|7 GB is associated with its suppressed mobility. The Mo 5|7 GB exhibits three distinct peaks centered at approximately 105, 112, and notably 160 nm. The high‐*D*_cum_ population near 160 nm indicates that some atoms in the Mo 5|7 core undergo substantially larger local rearrangements, which can facilitate dislocation migration. In contrast, the S 5|7 GB is concentrated mainly near 112 nm and shows no clearly resolved high‐*D*_cum_ population comparable to that of the Mo 5|7 GB. This lower displacement range indicates more restricted local atomic rearrangements, making migration of the S 5|7 core more difficult. This mobility contrast originates from the different local dynamic responses of the two dislocation cores. In Mo 5|7, the intrinsically high activity of site 3 is already present at room temperature and persists at 650°C, producing a preferentially activated rearrangement hotspot, whereas S 5|7 exhibits no comparable localized activation (Figure S10). A Mann–Whitney U test further confirmed that the *D*_cum_ value of site 3 in the Mo 5|7 core was significantly higher than those of all other sites (*p* < 10^−10^), whereas the S 5|7 core exhibited no comparably activated site. The relative migration tendencies predicted by the MD simulations are further supported by complementary first‐principles calculations (Figure S11). These calculations are intended to provide a qualitative comparison rather than quantitatively exact migration barriers, and they reproduce the lower glide barrier of Mo 5|7 relative to S 5|7.

In the mobile GB, site 3 is a key active site responsible for Mo 5|7 migration; this active site is absent in the immobile GB containing S 5|7. Building on this site‐resolved analysis, Figure 4d identifies the atomic pathways for Mo 5|7 glide and climb. The schematic in Figure 4di shows that both pathways originate from activation of site 3 and require coupled rearrangement of neighboring sites. In the glide pathway, site 3 moves cooperatively with site 4, producing lateral translation of the Mo 5|7 core, as confirmed by the corresponding in‐situ STEM snapshots in Figure 4dii. In the climb pathway, activation of site 3 is coupled with the removal of the neighboring site 2–3 atoms and subsequent bond reconstruction, which shifts the core vertically (Figure 4diii). These results show that Mo 5|7 migration proceeds through site‐3‐mediated glide and climb pathways, providing an atomistic mechanism for its high mobility.

## 3 Discussion

Our work establishes a direct structure‐kinetics‐growth relationship for GB evolution in polycrystalline monolayer MoS_2_ and our results show that GBs composed exclusively of Mo 5|7 dislocations are structurally smooth and kinetically mobile, whereas GBs containing S 5|7 dislocations develop kinked morphologies and become strongly pinned. At the microstructural level, mobile GBs drive rapid grain coarsening and GB elimination, while immobile GBs preserve a stagnant fine‐grained state. In‐situ heating STEM directly reveals that Mo 5|7 dislocations undergo thermally activated glide and climb in a correlated, chain‐like manner, whereas S 5|7 remains effectively immobile and interrupts the cooperative propagation of neighboring defects. MD simulations further identify a strongly activated internal site within the Mo 5|7 core, providing an atomistic explanation for its higher mobility, while the comparatively uniform and weaker local response of S 5|7 accounts for its pinning character.

Beyond clarifying the intrinsic kinetics of GB migration in monolayer MoS_2_, this work extends previous theoretical and computational understanding to direct experimental observation of site‐resolved dislocation dynamics and how dislocation chemistry governs microstructural evolution under realistic growth conditions. The h‐BN‐encapsulated in‐situ STEM platform demonstrated here provides a technical basis for resolving and tracking dynamic defect processes at the atomic scale under high‐temperature conditions. While h‐BN encapsulation may influence the absolute migration kinetics, the platform remains broadly useful for assessing relative trends in defect behavior, mobility, and collective GB dynamics under identical conditions. It should also be broadly useful for studying grain growth, defect healing, phase‐boundary motion, and interface stabilization. This perspective provides a basis for designing microstructures with tailored properties (e.g., mechanical strength and thermal transport) [41, 51] by controlling the mobility of specific defect units rather than treating GBs as structurally uniform interfaces.

## 4 Methods

### 4.1 Growth of Monolayer MoS_2_ by MOCVD

Growths were performed in a 4.5‐inch (inner diameter) hot‐wall quartz tube furnace. Glass substrates were rinsed with deionized water and blown dry using N_2_, then placed in the chamber. Molybdenum hexacarbonyl (Mo(CO)_6_) and diethyl sulfide ((C_2_H_5_)_2_S) were introduced to the chamber as Mo and S sources, respectively, together with H_2_ and Ar through separate lines. Samples were grown on Soda‐Lime and Alkali‐Aluminosilicate by flowing Mo(CO)_6_ (0.4 sccm), (C_2_H_5_)_2_S (3 sccm), H_2_ (3 sccm), and Ar (500 sccm) with a total pressure of 10 Torr at 650°C for 6, 12, 14, 18, and 24 h.

### 4.2 Growth of h‐BN Film by MOCVD

The growth of h‐BN films was conducted using c‐plane sapphire as substrates in an Aixtron G3 multiwafer planetary MOCVD reactor, which has the capacity to process 11 two‐inch wafers concurrently on its rotating susceptor. Growth conditions were established at 1050°C for the reactor temperature and 600 mbar for the chamber pressure. The boron and nitrogen sources were provided by triethylborane (TEB) and NH_3_, respectively, with H_2_ functioning as the carrier gas throughout the process. A pulsed source‐injection method was adopted to reduce unwanted gas‐phase parasitic reactions and ensure uniform layer‐by‐layer growth [52, 53]. The pulse sequence consisted of four distinct stages: (1) a 3–5 s TEB pulse; (2) a 1–2 s purge interval with precursor flow suspended; (3) a 1–4 s NH_3_ pulse; and (4) another 1–2 s purge interval. This cyclic process was repeated for a maximum of 100 iterations. TEB was supplied at low flow rates in the µmol/min regime (generally 10–20 µmol/min), while NH_3_ was introduced at 8,000 sccm, creating a significantly elevated V/III ratio that ensured nitrogen‐rich conditions. Throughout the h‐BN deposition process, the overall gas flow in the chamber remained fixed to maintain stable growth parameters and achieve uniform film quality over all substrates.

### 4.3 Transfer of MoS_2_ by Wet/Dry Transfer

To transfer MoS_2_ to the TEM grid, we spin‐coated poly‐methyl methacrylate (PMMA, 495K, 8A) on as‐grown samples at a speed of 1,000 rpm for 10 s and 4,000 rpm for 50 s with a spin coater at the Materials Imaging & Analysis Center of POSTECH in South Korea, then baked them at 100°C for 2 min. The edge of the coated film was scratched with a blade, then the film was gradually dipped into 10 vol.% HF with tilting. The PMMA/MoS_2_ stack was delaminated from the surface by water penetrating the interface, and then floated to the water surface. The film was scooped with a target TEM lacey carbon grid and baked at 100°C for 2 min to remove water and make conformal contact between MoS_2_ and the target surface. Finally, PMMA was removed by dipping it in acetone overnight and baked at 200°C overnight.

For in‐situ heating experiments, h‐BN/MoS_2_/h‐BN were assembled and transferred onto MEMS chips via a dry transfer process. Prior to transfer, a through‐hole type 4‐pin MEMS heating chip was cleaned by O_2_ plasma (10 W, 40 s) and baked in a vacuum oven at 80°C for 12 h. A thin Au layer was deposited on sapphire‐supported h‐BN using thermal evaporation, followed by attachment of thermal release tape (TRT) to the Au surface. The TRT/Au/h‐BN stack was detached from the sapphire substrate and placed onto the as‐grown MoS_2_ film. The stack was heated at 300°C for 10 min to improve adhesion, forming an Au/h‐BN/MoS_2_ structure. Using the TRT, this stack was then laminated onto another h‐BN flake and annealed again at 300°C for 10 min, producing a TRT/Au/h‐BN/MoS_2_/h‐BN structure. The entire stack was transferred onto a MEMS chip by dry stamping and heated at 150°C to release the TRT. Finally, the Au layer was selectively removed by wet etching, yielding a clean, fully encapsulated h‐BN/MoS_2_/h‐BN structure suitable for in‐situ STEM observation.

### 4.4 TEM and STEM Characterizations

For atomic resolution analysis of the crystal structure, STEM analyses (JEM‐ARM200F, JEOL) were performed at 80 kV. The instrument was equipped with a 5th order aberration corrector (ASCOR, CEOS GmbH) to form a 0.7 Å probe at Materials Imaging & Analysis Center of POSTECH in South Korea. The collection semi‐angles of the HAADF detectors ranged from 54 to 216 mrad. For in‐situ heating experiments, a DENS solutions heating system equipped with a through‐hole type 4‐pin MEMS heating chip mounted on a JEOL‐type 8‐pin double‐tilt heating holder was used. The heating sequence was controlled using the Impulse software, providing real‐time feedback and precise temperature regulation. In‐situ experiments were performed at 650°C under an accelerating voltage of 80 kV. Additional comparative observations were conducted at 550°C and 700°C. Time‐resolved in‐situ STEM movies were recorded at a resolution of 1,024 × 1,024 pixels with a dwell time of 8 µs per pixel, enabling direct atomic‐scale visualization of GB migration and dislocation motion under controlled thermal conditions.

### 4.5 STEM Movie Data Denoising

The in‐situ heating STEM movies were denoised using a self‐supervised deep learning framework based on the SHINE algorithm (Kim et al., Sci. Adv. 11, eads5552 (2025)) [35]. The method employs a blind‐spot convolutional neural network that statistically separates signal and noise within a single dataset without clean references, assuming that Poisson and electronic noise are spatially uncorrelated. Each recorded movie was divided into sequential frames, and the experimental noise correlation was analyzed to determine the optimal 5 × 5 blind‐spot kernel. The network predicted each pixel from its neighborhood while masking the center pixel, effectively removing random noise while preserving atomic contrast. Denoised frames were then temporally aligned and reconstructed into continuous movies for atomic‐scale analysis of dislocation motion and GB migration.

### 4.6 Defect Tracking Analysis

Defect migration was analyzed using an automated intensity‐based template‐matching procedure based on normalized cross‐correlation. Sequential in‐situ STEM images were treated as a time‐resolved dataset, and the initial position of the target defect was manually identified in the first frame. A feature region of interest (ROI) centered on the defect was extracted as a fixed reference template. The template contained both the defect core and the surrounding atomic‐column intensity pattern, thereby representing the local structural fingerprint of the defect. For each subsequent frame, template matching was performed within a larger search ROI centered on the defect position identified in the preceding frame. The reference template was compared with all candidate positions within the search ROI, and the position yielding the maximum normalized cross‐correlation coefficient was assigned as the defect position. Because the search was spatially restricted to the vicinity of the previously tracked position, no additional correlation threshold or failure criterion was applied. The dimensions of the feature and search ROIs were adjusted according to the image sampling and expected frame‐to‐frame displacement. The reference template was kept fixed throughout the image sequence to avoid cumulative template drift. The frame‐by‐frame defect coordinates were exported as a CSV file and converted from pixel units to physical distances, from which stepwise displacements, defect trajectories, and cumulative migration distances were calculated.

### 4.7 Fitting of Mobile and Immobile GB MoS_2_ Grain Growth

To quantify the relationship between grain growth rate and GB mobility, the average grain sizes for both samples were fitted to the parabolic grain growth law:

(1) $$D^{n}-\;{D_{0}}^{n}=Kt$$

where *D* and *D*_0_ represent the average grain size at time t and the initial size, *K* is the growth rate constant dependent on GB mobility and interfacial energy, and n is the growth exponent. The best‐fit exponent n = 1.98 for the mobile GB—MoS_2_ indicates continuous grain growth governed by active GB migration, whereas n = 0 for the immobile GB MoS_2_ reflects complete stagnation due to suppressed GB migration (Table S2) [54]. The rate constant K = 0.152 µm^1^·^9^ ^8^/h for the mobile GB MoS_2_ further confirms that grain coarsening proceeds through thermally activated GB migration, while the immobile‐GB MoS_2_ shows no measurable K, consistent with its pinned GB structure. Together, these results demonstrate that macroscopic grain growth in monolayer MoS_2_ is directly governed by GB mobility [39, 40].

Grain sizes were quantified from the original DF‐TEM images using ImageJ. After spatial calibration, FFT band‐pass filtering and intensity thresholding were applied to generate binary grain masks, which were subsequently inspected and manually corrected to ensure accurate GB delineation. The projected area of each grain was measured using the Analyze Particles function and converted to an equivalent grain radius. Grains intersecting the image boundaries were excluded because their complete projected areas could not be determined. At each growth time of 6, 12, 14, 18, and 24 h, 50 grains associated with mobile GBs and 50 grains associated with immobile GBs were analyzed. The data points in Figure 1c represent the mean equivalent grain radius, and the error bars indicate one standard deviation of the corresponding grain‐radius distribution at each growth time.

### 4.8 Molecular Dynamics Simulations of MoS_2_ With GB

The preparation of GB‐containing single‐layer MoS_2_ (SLMoS_2_) structures was carried out using MD simulations implemented in the LAMMPS package [55]. The initial configurations were constructed with embedded Mo‐ and S‐centered 5|7 dislocation arrays representing twin GBs. The interatomic interactions were described using a MoS_2_‐specific reactive empirical bond‐order (REBO) potential [29, 56] that has been demonstrated to reliably capture the mechanical properties of MoS_2_‐based nanostructures. Although empirical potentials may involve some uncertainty in absolute barrier values, the present REBO potential is appropriate for large‐scale comparative analyses of dislocation‐core dynamics under identical simulation conditions. The simulation cell contained more than 65,000 atoms, allowing extended dislocation structures and their collective atomic responses to be examined with sufficient statistical sampling. Statistical comparisons of the site‐resolved *D*_cum_ distributions were performed using the Mann–Whitney U test because the distributions deviated from normality.

Prior to MD simulations, the structures were first relaxed via energy minimization. Subsequently, the systems were equilibrated through MD simulations for 1 ns at 300 K and zero pressure in the isothermal–isobaric (NPT) ensemble. The atomic motions were integrated using the velocity‐Verlet algorithm with a time step of 1.0 fs. Temperature and pressure were controlled using the Nosé–Hoover thermostat and barostat with damping constants of 100 and 1,000 time steps, respectively. During the equilibration at 300 K, the trajectories were recorded for post‐analysis.

The cumulative non‐affine displacement *D*_cum_ is evaluated to quantify local atomic rearrangements during MD simulations. This quantity is defined as the summation of the square roots of the incremental non‐affine displacements $\Delta D_{i,\min}^{2}$ over successive time intervals, and has been widely employed to characterize dynamic heterogeneity and atomic mobility in disordered systems [57, 58]. Following previous studies [59, 60], the non‐affine displacement $\Delta D_{i,\min}^{2}$ between time *t* and *t* + Δ*t* is calculated as:

(2) $$D_{\min}^{2}=\frac{1}{M_{k}}\;\sum_{i}^{M_{k}}{\left[r_{\mathrm{ik}}\left(t+\Delta t\right)-J_{k}\left(t\right)r_{\mathrm{ik}}\left(t\right)\right]}^{2}$$

where ***r***_ik_(*t*) denotes the relative position vector between atom *i* and *k* at time *t*, ***J***_k_(*t*) is the local strain tensor about atom *k* that minimizes $D_{\min}^{2}\left(k;t\right)$, *M*_k_ is the total number of neighboring atoms around atom k.

(3) $$D_{\mathrm{cum}}=\sum_{i=1}^{n}\sqrt{\Delta D_{i,\min}^{2}}\;$$

where $\Delta D_{i,\min}^{2}$ is the increment of $D_{min}^{2}$ with Δ*t*, and n is the total number of intervals during the MD simulations at 300 K. The simulation protocol was initially optimized at 300 K and subsequently applied under identical settings at 923 K to assess the defect dynamics at the experimentally relevant temperature.

### 4.9 DFT Calculations of Glide Energy Barriers

First‐principles calculations were performed using the CASTEP module of the Materials Studio software with the Perdew‐Burke‐Ernzerhof (PBE)‐GGA, ultrasoft pseudopotentials, and the energy cutoff of 299.3 eV. The glide pathways of the Mo 5|7 and S 5|7 dislocation cores were examined using the complete linear synchronous transit/quadratic synchronous transit (LST/QST) method implemented in the TS Search module. The energy difference between the initial structure and the maximum‐energy structure along the LST path was used to estimate the relative glide energy barrier.

## Author Contributions

C.‐W.C. and S.‐Y.C. conceived and designed the project. C.‐W.C. and S.‐Y.C. carried out the (S)TEM imaging and analysis. M.‐Y.C. and C.‐J.K. fabricated the monolayer MoS_2_ samples. T.D. and X.L. conducted the MD simulations. S.M. and J.K.K. provided the 2 nm‐thick h‐BN encapsulation layers. J.R. and J.P. denoised the in‐situ movie data. Y.‐S.N. and S.‐Y.C. performed first‐principles calculations. C.‐W.C. and S.‐Y.C. analyzed all data and wrote the manuscript. All authors discussed the results and commented on the manuscript.

## Conflicts of Interest

The authors declare no conflicts of interest.

## Supporting information




**Supporting File 1**: advs77465‐sup‐0001‐SuppMat.pdf.

**Supporting File 2**: advs77465‐sup‐0002‐MovieS1.mp4.

**Supporting File 3**: advs77465‐sup‐0003‐MovieS2.mp4.

## Data Availability

The data that support the findings of this study are available from the corresponding author upon reasonable request.
