# Supplementary material for: Atomistic Kinetics of Dislocation‐Mediated Grain Growth in Monolayer MoS2
Source: Adv Sci (Weinh). 2026 Aug 30:e77465. Online ahead of print. doi: 10.1002/advs.77465 (PMC13526703; doi:10.1002/advs.77465)
Supplement: Supplementary file 1 [file ADVS-9999-e77465-s003.pdf]

Supporting Information

Atomistic Kinetics of Dislocation-Mediated Grain Growth in Monolayer MoS_2

Chang-Won Choi[1,2,†], Min-Yeong Choi[1,3,†], Tao Du[4], Jinho Rhee[5], Seokho Moon[2,6,7], Yeon-Seo Nam[2], Xiaoyan Lu[4], Jong Kyu Kim[2], Jungwon Park[5], Moon-Ho Jo[1,2,8], Cheol-Joo Kim[1,3], and Si-Young Choi[1,2,9]**

[1] Center for Van der Waals Quantum Solids, Institute for Basic Science (IBS), Pohang 37673, Republic of Korea.

[2] Department of Materials Science and Engineering, Pohang University of Science and Technology (POSTECH), Pohang 37673, Republic of Korea.

[3] Department of Chemical Engineering, POSTECH, Pohang 37673, Republic of Korea.

[4] School of Civil Engineering, Harbin Institute of Technology, Harbin 150001, China.

[5] Department of Chemical and Biological Engineering, Seoul National University (SNU), Seoul 08826, Republic of Korea.

[6] Department of Materials Science and Engineering, Ajou University, Suwon 16499, Republic of Korea.

[7] Department of Energy Systems Research, Ajou University, Suwon 16499, Republic of Korea.

[8] Department of Physics, POSTECH, Pohang 37673, Republic of Korea.

[9] Department of Semiconductor Engineering, POSTECH, Pohang 37673, Republic of Korea.

* Correspondence to S.-Y. Choi (youngchoi@postech.ac.kr) and C.-J. Kim (kimcj@postech.ac.kr).

[†]These authors contributed equally to this work.

Table of Contents

Supporting Text

Note S1: Crystallographic definitions of the Burgers vectors and GB vectors.

Note S2: Crystallographic definitions of the GB inclination angle and GB planes.

Supporting Figures

Figure S1. SAED patterns and corresponding DF-TEM images of mobile and immobile GB – MoS_2 grains.

Figure S2. Grain-size distributions of grains associated with mobile and immobile GBs during the growth of monolayer MoS_2.

Figure S3. Growth temperature condition and MoS_2 film morphology.

Figure S4. Quantitative analysis of GB defect.

Figure S5. Structural dependence of MoS_2 GBs on the inclination angle (ϕ).

Figure S6. Atomic-position validation of the denoising process.

Figure S7. Cumulative distance of dislocations in mobile and immobile GBs.

Figure S8. Representative HAADF-STEM images of mobile- and immobile-type GB configurations observed after heating at 550 and 700 °C.

Figure S9. MD simulation models of Mo 5|7 and S 5|7 GBs.

Figure S10. Site-dependent local dynamic responses of Mo 5|7 and S 5|7 at 300 K obtained from MD simulations.

Figure S11. First-principles calculations of the glide energy barriers for Mo 5|7 and S 5|7 dislocations.

Supporting Table

Table S1. Statistical figures of grain size changes during the growth time of mobile and immobile $GB-MoS_2$.

Table S2. Fitted constants by the parabolic growth law of mobile and immobile GB -MoS_2.

Supporting Text

Note S1: Crystallographic definitions of the Burgers vectors and GB vectors.

Figure 2 compares the crystallographic atomic structures of mobile and immobile GBs in monolayer MoS_2. Figure 2a defines a mobile GB composed exclusively of Mo 5|7s between two misoriented grains with a crystallographic tilt angle (θ_t). The Burgers vector of Mo 5|7 ($\vec{b}_{(1,0)}$, a purple arrow) is determined by a Burgers circuit, which traces a black dashed line of clockwise loop around the Mo 5|7. The GB vector, $\overrightarrow{GB}_{(-1,2)}$, corresponds to the GB's crystallographic orientation. Figure 2d defines the atomic structure of the immobile GB composed of Mo 5|7 and S 5|7. The orange-colored Burgers vector of S 5|7 ($\vec{b}_{(0,1)}$) is defined in addition to that of Mo 5|7 ($\vec{b}_{(1,0)}$) by a Burgers circuit, which traces a loop around the S 5|7. The introduction of S 5|7 results in a shift of the GB vector from $\overrightarrow{GB}_{(-1,2)}$ to $\overrightarrow{GB}_{(-2,1)}$.

Note S2: Crystallographic definitions of the GB inclination angle and GB planes.

Although the mobile and immobile GBs have a similar crystallographic θ_t of 22°, their local boundary orientations are different. To distinguish this difference, we define the inclination angle (ϕ) as the angle between the local GB direction and the reference GB axis. The mobile GB maintains a nearly straight boundary direction with ϕ = 0°, whereas the immobile GB contains inclined boundary segments with ϕ = 60° (Figure S5a-b). Thus, the two GBs have comparable grain misorientation but different local GB inclinations. This difference in ϕ reflects the different dislocation-core arrangements along the GB. In the mobile GB, periodically aligned Mo 5|7 dislocations lie along the reference GB axis, producing a straight and structurally uniform boundary (Figure S5a & c). The corresponding HAADF-STEM image confirms that the mobile GB maintains ϕ = 0° while preserving a θ_t of 22°. In contrast, the immobile GB contains both Mo 5|7 and S 5|7 dislocations, and the incorporation of S 5|7 locally rotates the GB direction away from the reference axis. This produces inclined segments with ϕ = 60°, resulting in a zig-zag or kinked GB morphology (Figure S5b & d).

Crystallographic plane indexing provides the same interpretation. The mobile GB forms a continuous symmetric $(140)_L/(\bar{1}40)_R$ interface, where the two 22°-tilted grains meet through crystallographically equivalent high-index planes. This symmetric plane matching allows the GB to remain straight. By contrast, the immobile GB follows low-index planes of the left grain that connect to higher-index planes of the right grain, forming asymmetric $(1\bar{1}0)_L/(140)_R$ and $(010)_L/(310)_R$ facets. These asymmetric plane junctions change the local ϕ and create kink sites. Therefore, the immobile GB is not defined simply by a different tilt angle, but by local changes in GB inclination and asymmetric plane matching induced by S 5|7 dislocations.

In summary, the mobile GB is characterized by a uniform Mo 5|7 array, a straight boundary direction (ϕ = 0°), and symmetric plane matching. The immobile GB is characterized by mixed Mo 5|7 & S 5|7 cores, inclined GB segments (ϕ = 60°), asymmetric plane matching, and kinked morphology. These crystallographic differences provide the structural basis for the mobile and immobile GB behaviors discussed in the main text.

Supporting Information

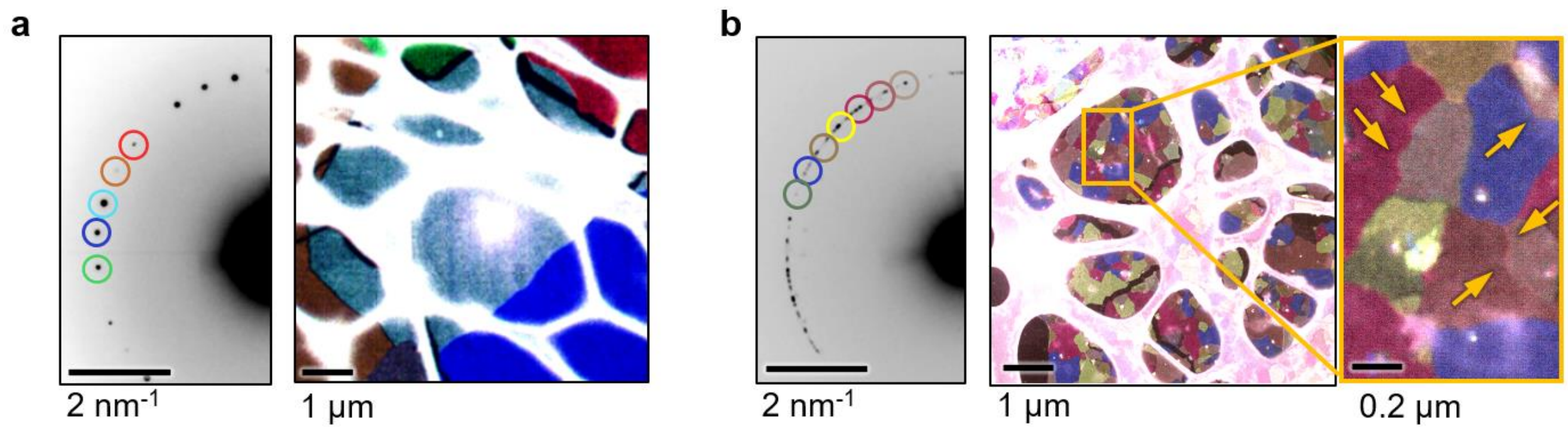

Figure S1. SAED patterns and corresponding DF-TEM images of mobile and immobile GB – MoS_2 grains. a) SAED pattern and corresponding DF-TEM image of MoS_2 with mobile GBs. b) SAED pattern and corresponding DF-TEM image of MoS_2 with immobile GBs. Colored circles in the SAED patterns correspond to the crystallographic orientations of the grains shown in the DF-TEM images, where each color represents a distinct orientation.

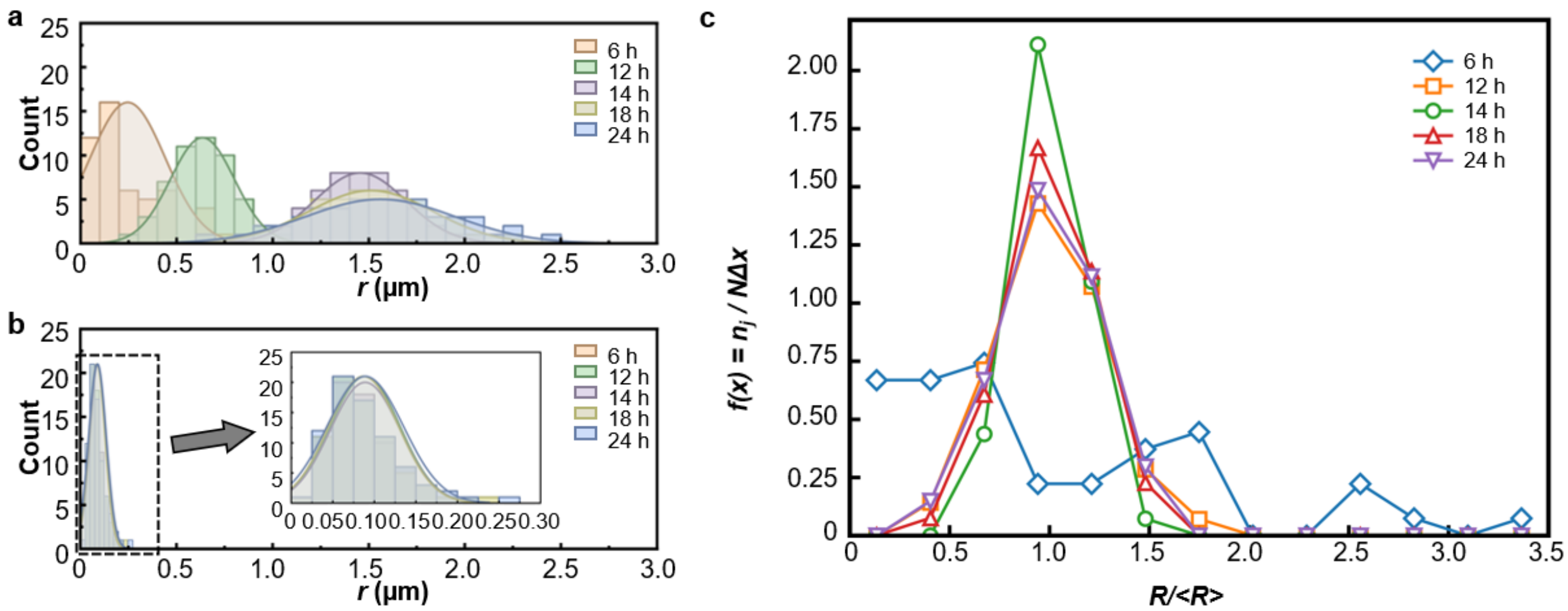

Figure S2. Grain-size distributions of grains associated with mobile and immobile GBs during the growth of monolayer MoS_2. a) Grain-radius distributions of grains associated with mobile GBs MoS_2 after 6 h (orange), 12 h (green), 14 h (purple), 18 h (olive), and 24 h (blue) of growth time. The solid curves represent fitted distribution profiles for each growth time. b) Corresponding grain-radius distributions for grains associated with immobile GBs MoS_2. The inset enlarges the low-radius region to highlight the overlapping distributions. c) Scaled grain-size distributions (f(x)) of grains associated with mobile GBs as a function of the normalized grain radius (R/<R>).

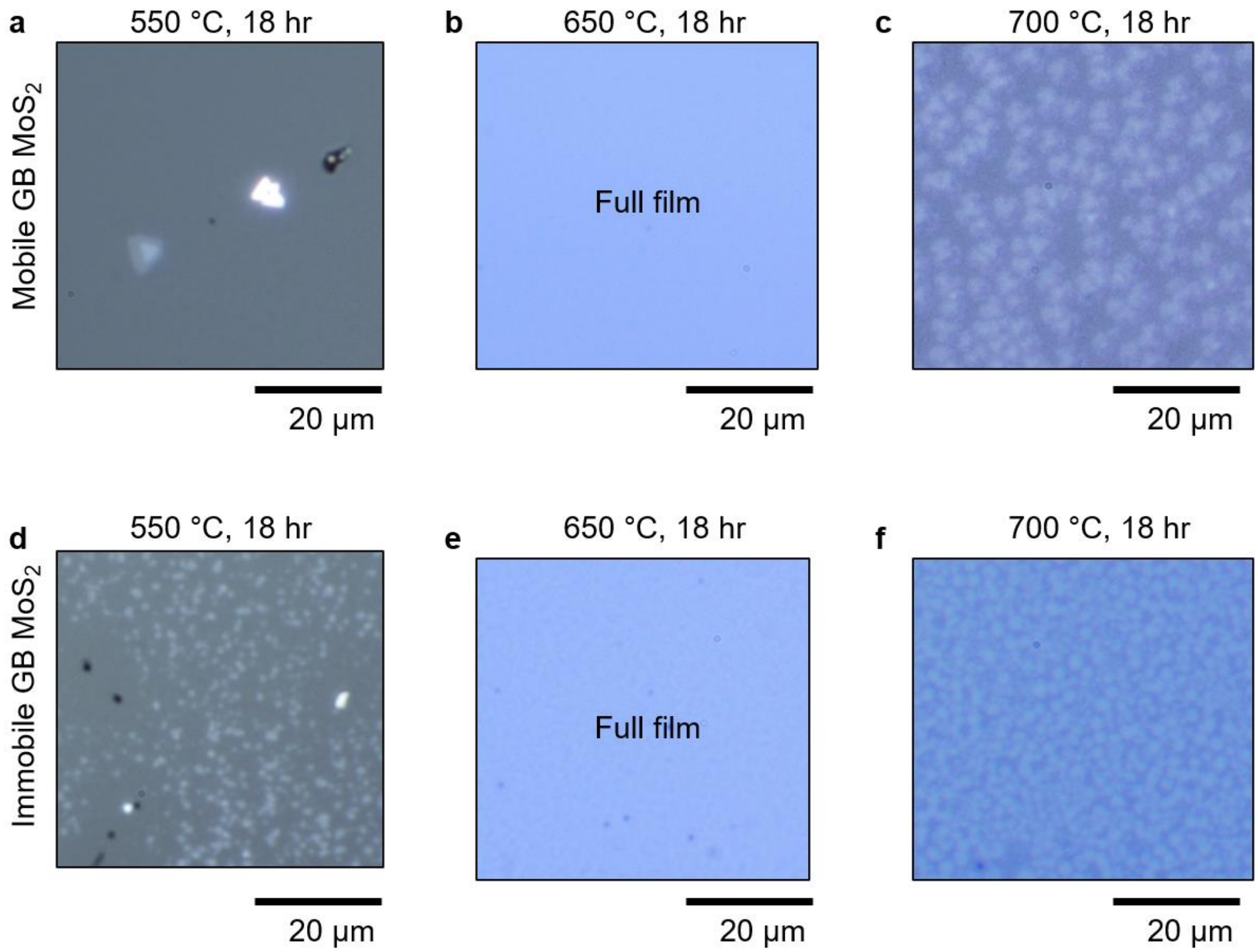

Figure S3. Growth temperature condition and MoS_2 film morphology. Optical microscopy images of mobile and immobile MoS_2 synthesized by MOCVD at 550, 650, and 700 °C for 18 h. (a-c) Mobile MoS_2 and (d-f) immobile MoS_2.

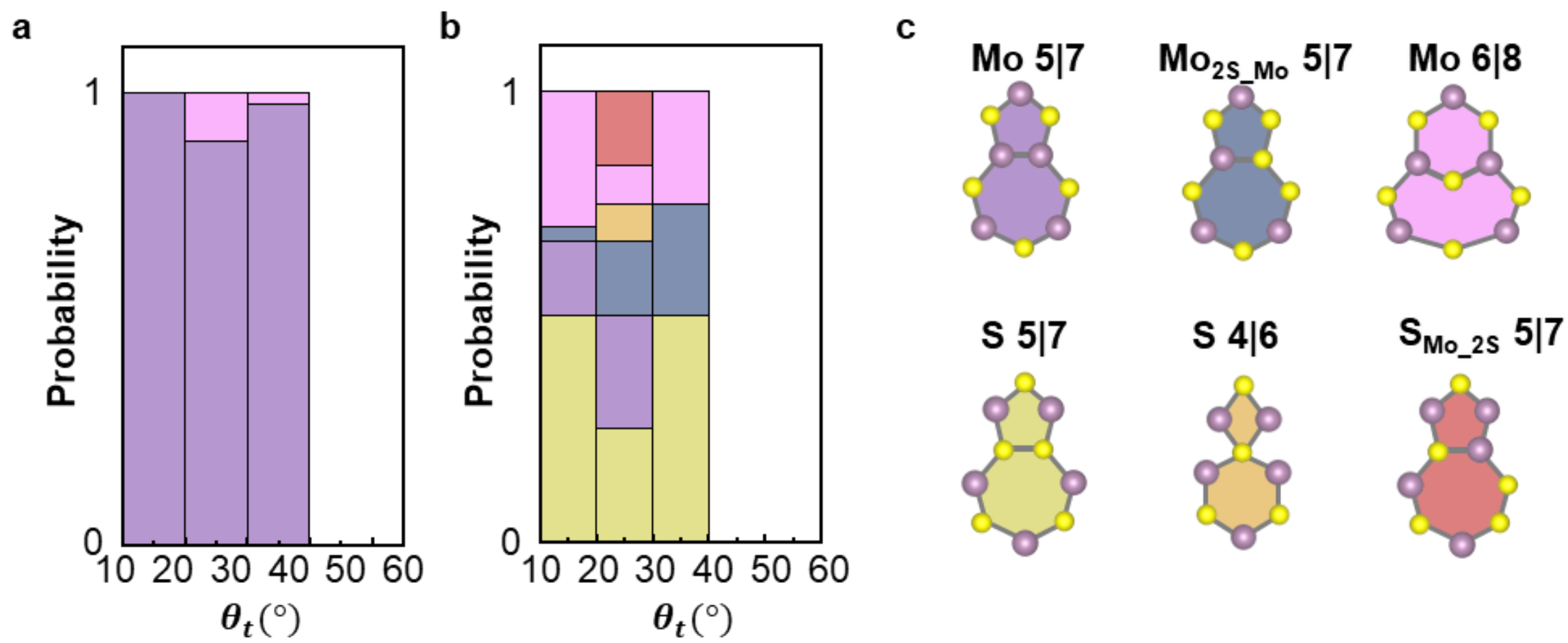

Figure S4. Quantitative analysis of GB defect. a) Quantitative analysis of GB defect of mobile GB-MoS_2. b) Quantitative analysis of the GB defect of immobile GB-MoS_2. c) GB defect types. Defect types shown in each color correspond to the graphs of (a) and (b).

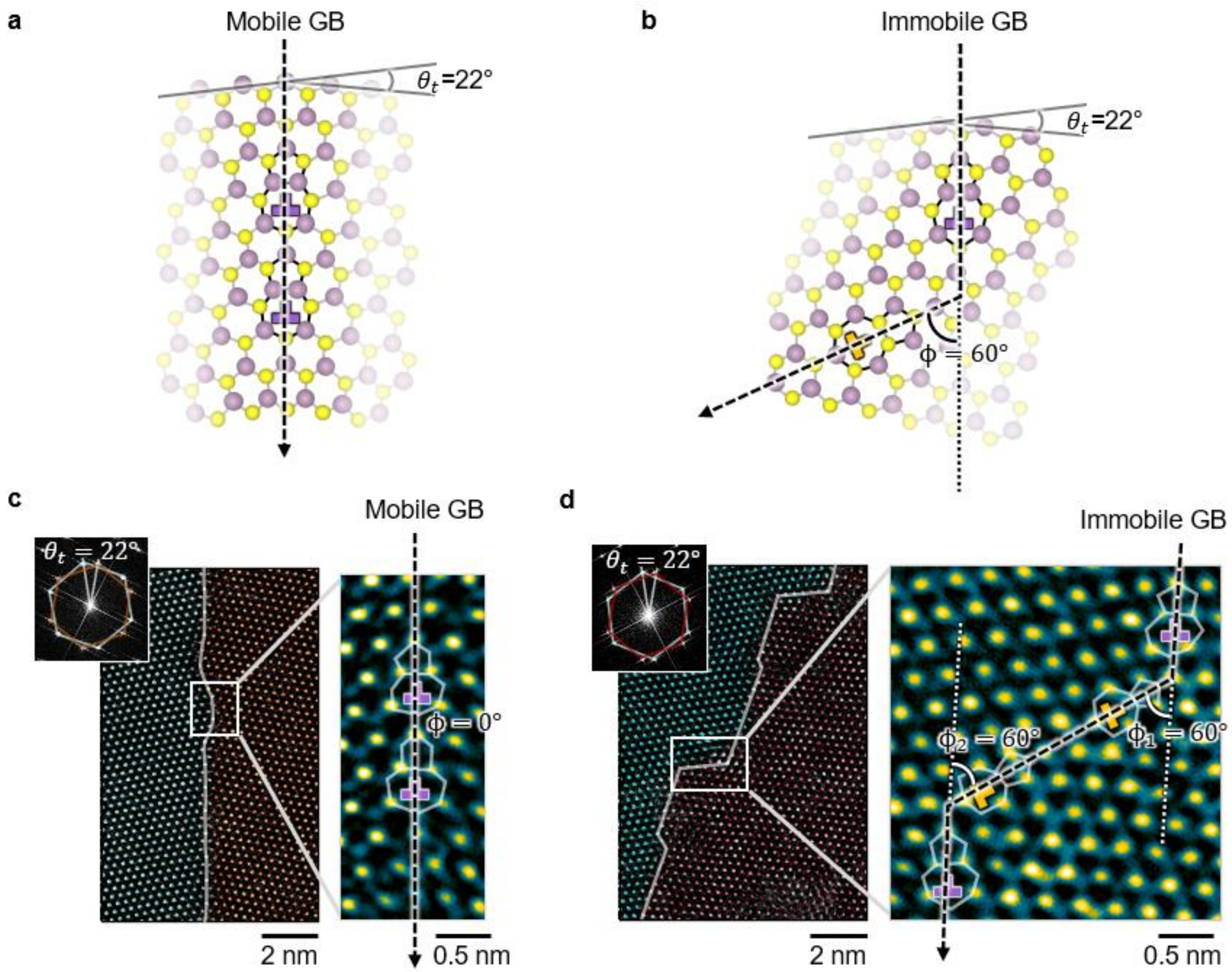

Figure S5. Structural dependence of MoS_2 GBs on the inclination angle (ϕ). a) Schematic illustration θ_t= 22° GBs of mobile GB with $\phi = 0°$, and b) immobile GB with $\phi = 60°$. Black dashed arrows represent the GB, and dotted line shows the reference GB axis. The purple and orange ⊥ represent the dislocation of Mo 5|7 and S 5|7, respectively. c) HAADF–STEM images of GBs of mobile GB with $\phi = 0°$ (FFT indicated θ_t= 22°) and d) immobile GB ($\phi = 60°$) which exhibits a zig-zag. The magnified views in (c) and (d) demonstrate that the different inclination angles lead to distinct configurations of the 5|7 dislocation cores.

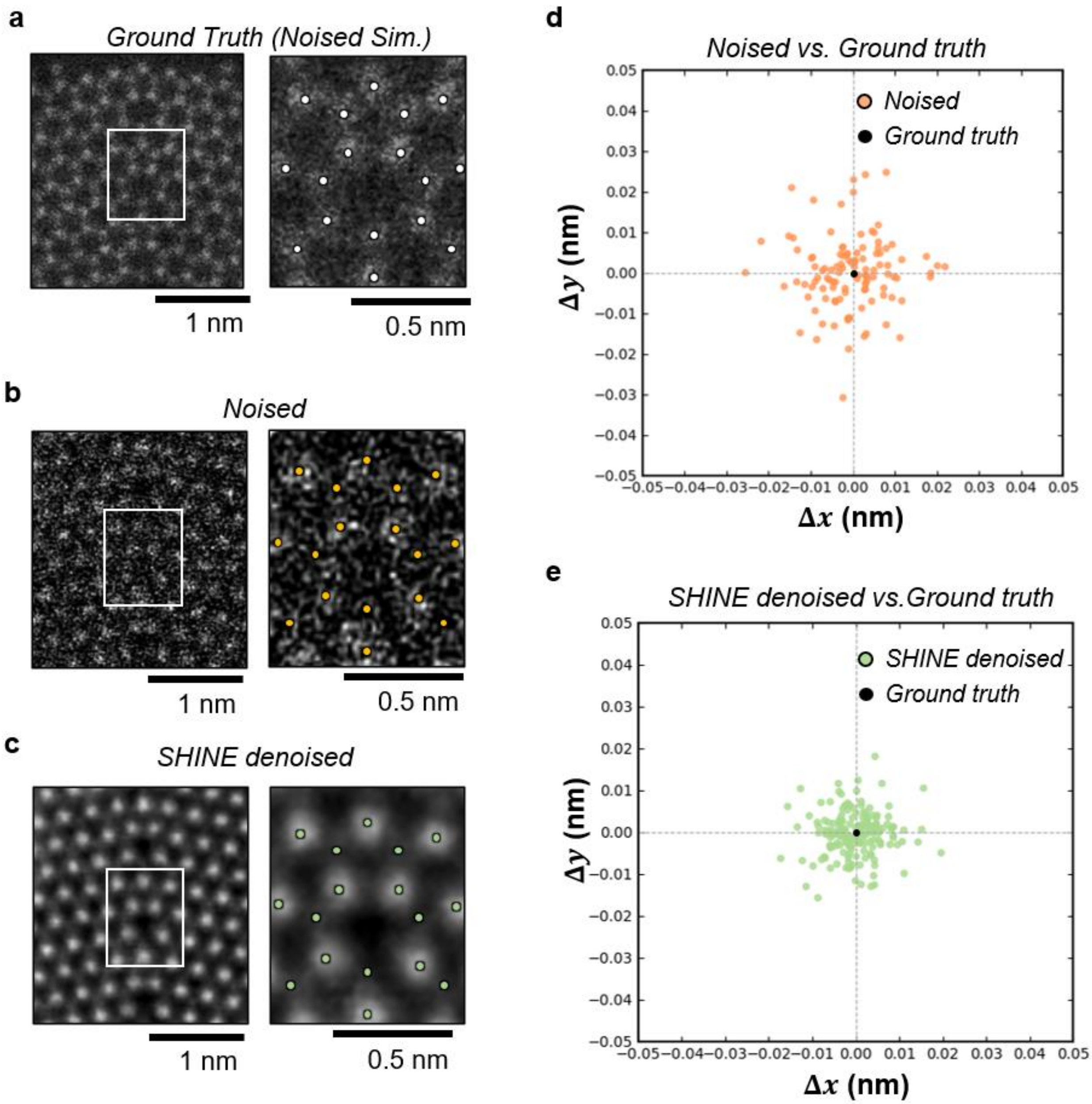

Figure S6. Atomic-position validation of the denoising process. (a) Simulated HAADF-STEM image and corresponding atomic positions. (b) Noised experimental HAADF-STEM image and detected atomic positions. (c) SHINE-denoised image and detected atomic positions. (d) Positional deviations between the raw image and the ground-truth simulation. (e) Positional deviations between the SHINE-denoised image and the ground-truth simulation.

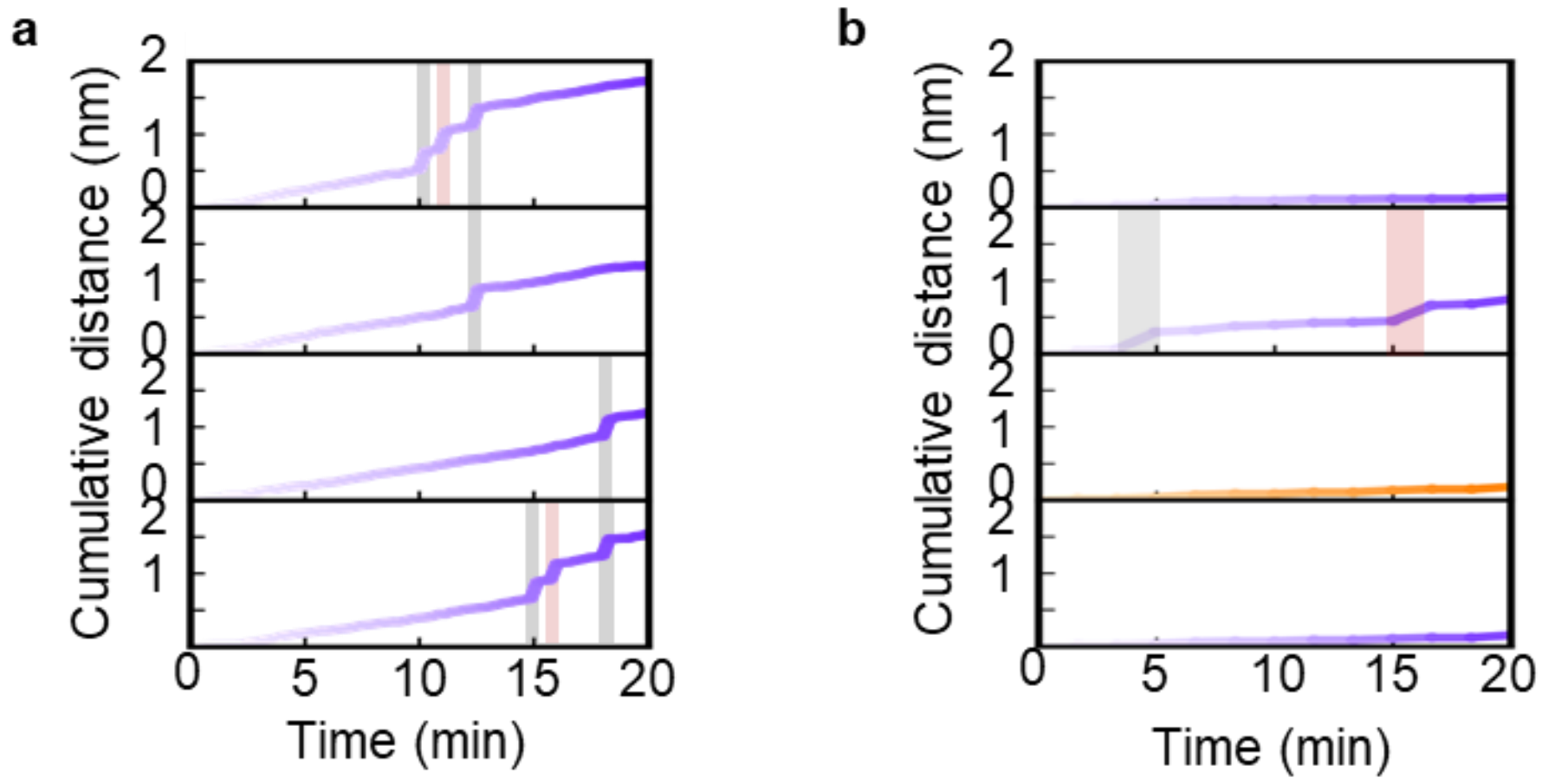

Figure S7. Cumulative distance of dislocations in mobile and immobile GBs. a) Cumulative distance of individual Mo 5|7s. Grey regions indicate forward climbing/gliding, while red regions indicate backward climbing/gliding. b) Cumulative distance of individual Mo 5|7 and S 5|7. Grey regions indicate forward gliding.

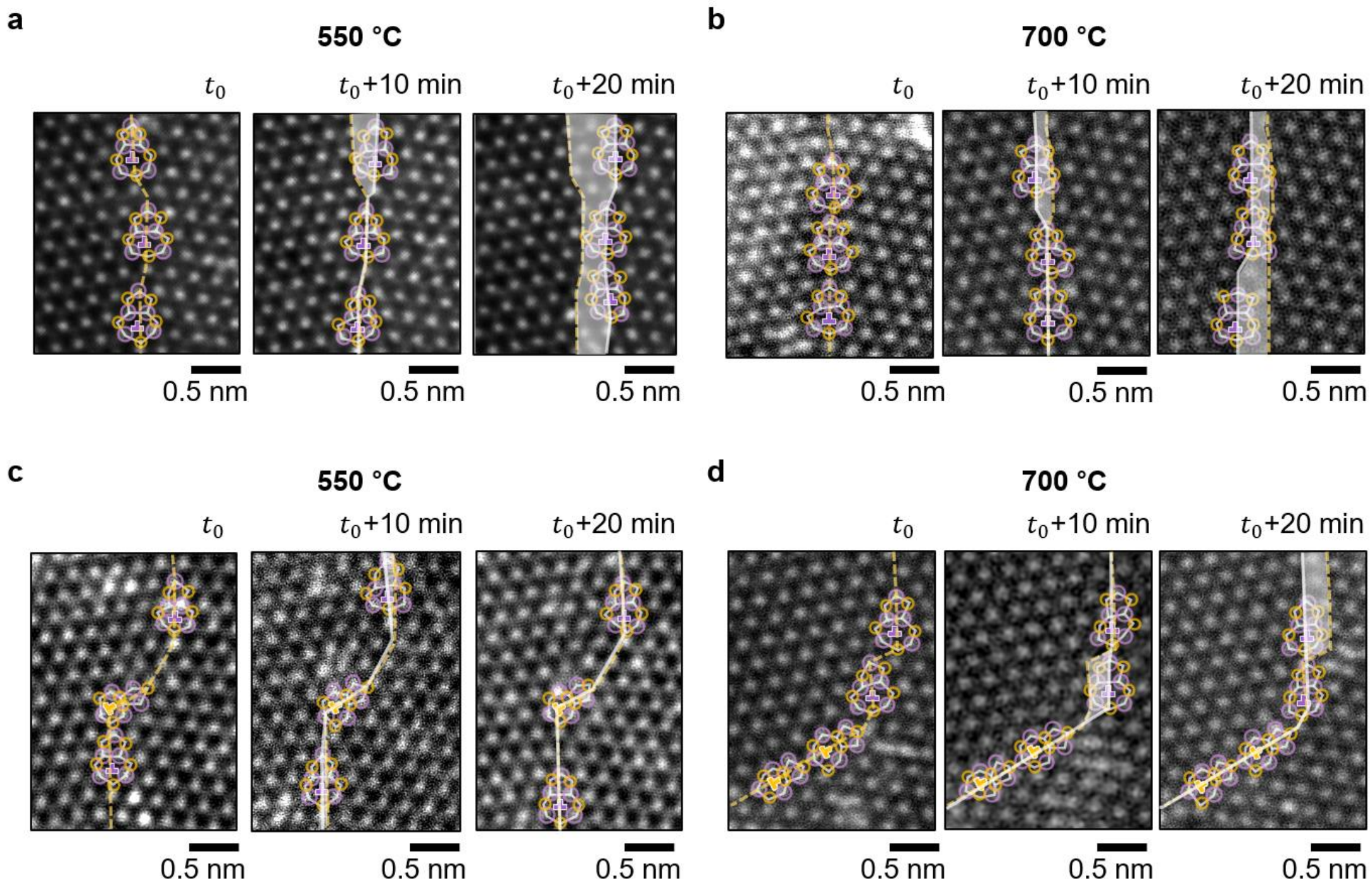

Figure S8. Representative HAADF-STEM images of mobile- and immobile-type GB configurations observed after heating at 550 and 700 °C. a) Mobile GB evolution at 550 °C. b) Mobile GB evolution at 700 °C. c) Immobile GB evolution at 550 °C. d) Immobile GB evolution at 700 °C.

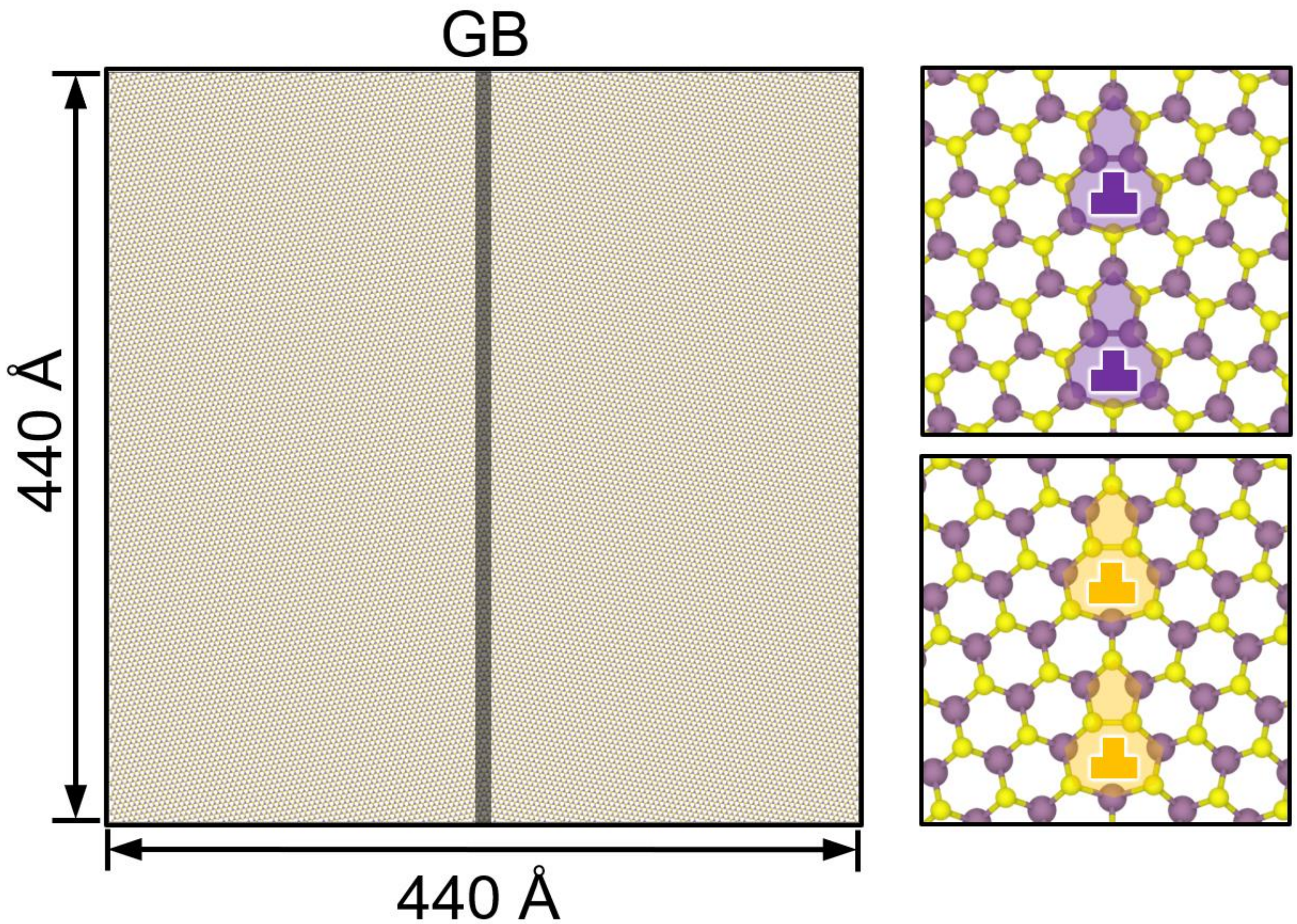

Figure S9. MD simulation models of Mo 5|7 and S 5|7 GBs.

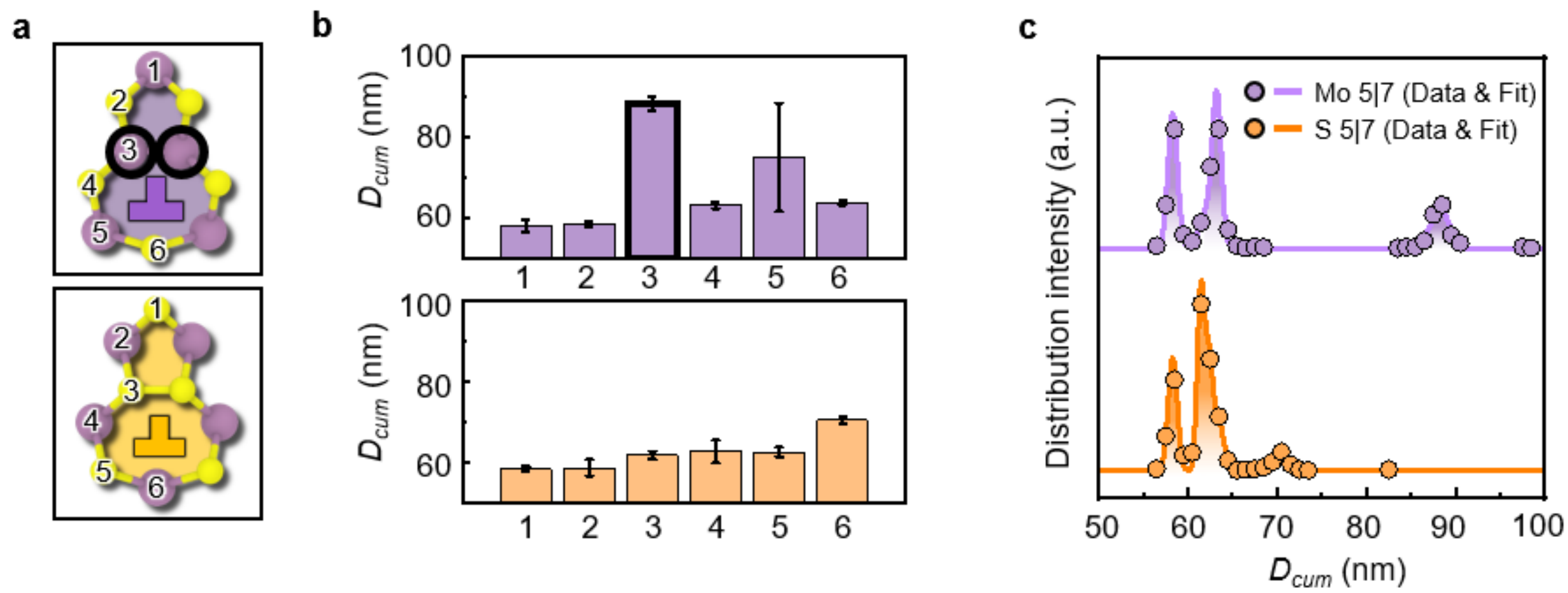

Figure S10. Site-dependent local dynamic responses of Mo 5|7 and S 5|7 at 300 K obtained from MD simulations. a) Schematics defining the atomic sites (1-6) within the Mo 5|7 (upper panel) and S 5|7 (lower panel). The highlighted site indicates the atomic positions exhibiting enhanced local dynamic activity. b) Site-resolved D_{cum} values for atoms located at different positions within the GB structures. The upper and lower panels correspond to the Mo 5|7 and S 5|7 dislocation cores, respectively. c) Distributions of D_{cum} for atoms within different GBs after 1 ns of simulation.

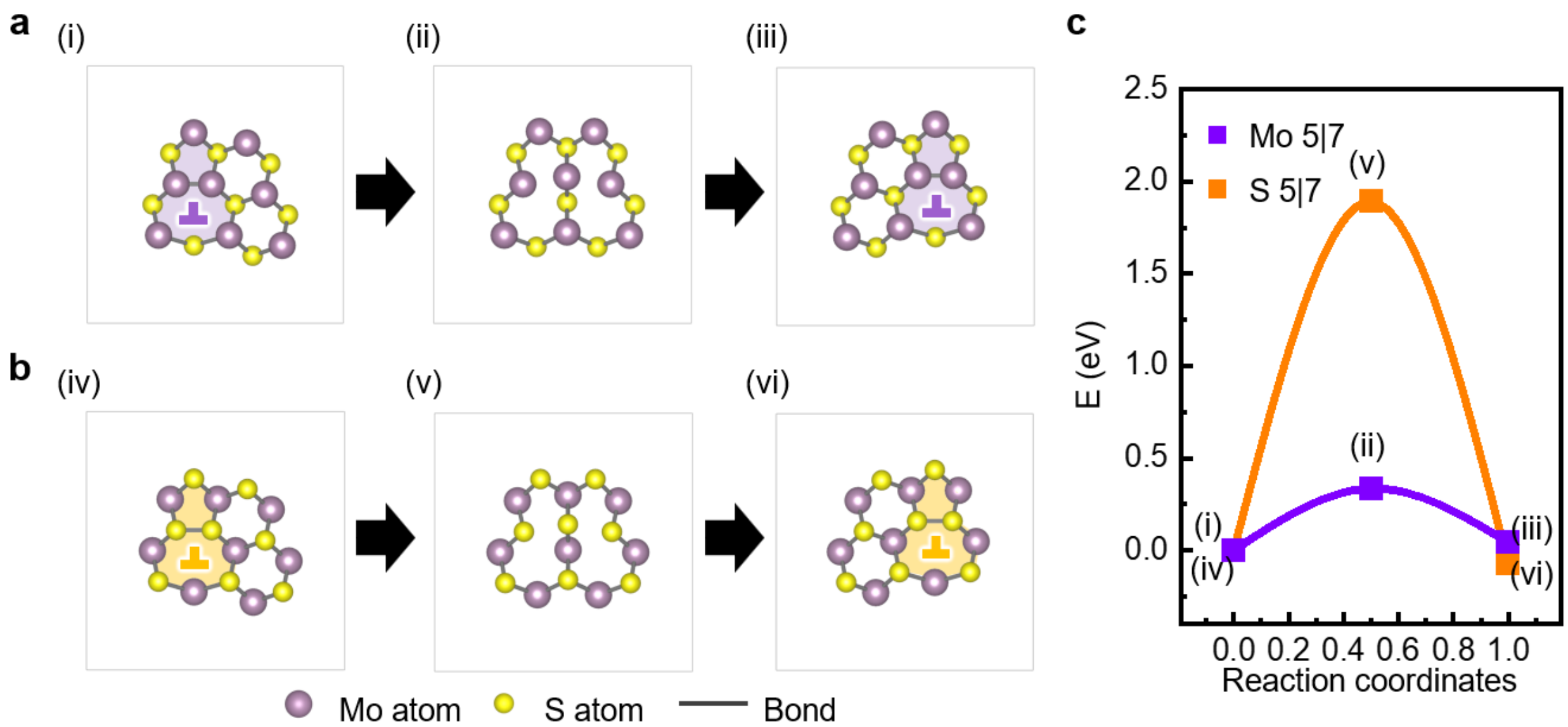

Figure S11. First-principles calculations of the glide energy barriers for Mo 5|7 and S 5|7 dislocations. a) Glide mechanism of the Mo 5|7 dislocation. b) Glide mechanism of the S 5|7 dislocation. c) Calculated relative glide energy barriers for the Mo 5|7 and S 5|7 dislocations.

Supporting Table

Growth time (hr)	Mobile GB - MoS_2		Immobile GB-MoS_2	
	r_M (µm)	Standard deviation	r_I (µm)	Standard deviation
6	0.24	0.20	0.087	0.042
12	0.63	0.17	0.089	0.042
14	1.45	0.24	0.089	0.042
18	1.51	0.32	0.087	0.042
24	1.56	0.39	0.089	0.046

Table S1. Statistical figures of grain size changes during growth time of mobile and immobile GB-MoS_2.

	n	**K**	R^2
Mobile GB - MoS_2	1.98	0.152 $\mu m^{1.98}/hr$	0.82
Immobile GB - MoS_2	0	null	-

Table S2. Fitted constants by parabolic growth law of mobile and immobile GB -MoS_2.
